# Exploring the illness representations of people with psoriatic arthritis: a secondary analysis of focus group data

**DOI:** 10.1093/rap/rky023

**Published:** 2018-08-08

**Authors:** George Erskine, Emma Dures, Neil McHugh, Sarah Hewlett

**Affiliations:** 1 Academic Rheumatology, University of the West of England, Bristol Royal Infirmary, Bristol, UK; 2 Department of Pharmacy and Pharmacology, University of Bath, Bath, Somerset, UK

**Keywords:** spondylarthropathies, rheumatic diseases, illness representation, common-sense model, patient attitude to health, behaviour, psychology and social phenomena

## Abstract

**Background:**

PsA is an inflammatory arthritis associated with psoriasis, affecting the joints and connective tissue. According to the common-sense model (CSM), patients develop illness beliefs when faced with new symptoms, which determine their emotional and behavioural response to the condition. The CSM includes five beliefs: identity, consequence, cause, time line and control. These are important determinants of outcomes and have been shown to influence adherence to medication.

**Methods:**

A secondary analysis of eight focus groups held across five hospital sites and including 41 participants was undertaken. Participants were sampled for a range of phenotypes and domains of disease activity: men = 20 and women = 21; mean (s.d.): age = 58 (11.4) years; disease duration = 9 (8.3) years; and HAQ = 1 (0.7).

**Results:**

The analysis provided evidence to support the existence of the five illness beliefs among patients with PsA and evidence that these representations affected the way patients engaged with their condition. The results showed that many participants experienced high levels of uncertainty in relationship to the illness representation. The role of external social and environmental factors was also shown to influence participants’ illness representations and the way they made sense of their PsA.

**Conclusion:**

This research highlights a new theme of uncertainty within illness representations and how this impacts on the way in which people living with PsA manage their condition. A greater understanding of the uncertainty that patients might have about their condition and its treatment could provide clinicians with an opportunity to address misinformed illness representations.

**Key messages**

- Within this study uncertainty has been shown to play a large part in illness representations and how people living with PsA manage their condition.

## Introduction

The prevalence of psoriasis within the UK is estimated at 2–3% of the population, with ≥30% of those developing PsA [1]. PsA is an inflammatory arthritis affecting the joints and connective tissue and is usually associated with psoriasis [1, 2]. In the long term, the condition causes progressive joint damage [1, 3]. The main clinical features include: enthesitis (inflammation at tendon/ligament attachments), dactylitis (digit swelling), spondylitis (inflammatory back pain) and inflammation of DIP joints [4]. In addition to the physical burden of the illness, many patients with PsA also experience high levels of anxiety and depression associated with psychological distress, which has been shown to remain high even when disease severity is low [3].

It has been proposed that patients develop representations (lay beliefs) of their illness to make sense of, and manage, their condition [2]. Illness representations are based on the Leventhal’s common-sense model (CSM), which includes five domains: identity (the label/diagnosis given to the condition and the symptoms), time line (beliefs about how long the condition might last), consequence (beliefs about the consequence of the condition), cause (beliefs about the cause of the condition) and control (beliefs about whether the condition can be cured or managed) [5, 6]. Leventhal suggested that when patients are faced with new symptoms or a diagnosis, they will initiate the development of representations that will influence their emotional and behavioural coping strategies. Previous research in a range of long-term conditions, including diabetes, chronic fatigue syndrome, irritable bowel syndrome and psoriasis, has demonstrated a link between the CSM of illness representations and the way in which people make sense of and manage their condition [7].

Although there has been limited research regarding the representations that people with PsA hold concerning their condition and how they affect outcomes, the available research suggests that the CSM is a useful framework for gaining insight into patients’ responses to PsA and its treatment. Representations can be informed by a range of sources and influenced by personal and contextual factors. As such, they have the potential to be erroneous or unhelpful, with evidence suggesting that they can lead to negative coping strategies, including social isolation, alcoholism and psychological distress [3, 6, 8]. Quantitative research has also linked illness representations to poor health-related quality of life (QoL) and psychological distress through numerous concerns regarding bodily symptoms (identity) and the perceived negative consequences of PsA [2, 9].

Representations have been shown to impact on patients’ behavioural and psychological responses to their condition; for example, a perception that the illness is controllable was positively related to patients’ self-management, well-being and social functioning [7]. As a consequence, representations are important determinants of outcome [2, 7].

In a previous qualitative study to identify treatment outcomes important to patients with PsA, participants in focus groups discussed minimizing harm and burden from the condition and from the treatment, alongside reducing the impact and striving to optimize their prognosis [10]. Participants’ accounts of their treatment-related decisions and behaviours were set in the context of their underlying representations about PsA. Insights into these representations could help clinicians to understand patients’ responses to their PsA and their treatment decisions. Furthermore, they might also help clinicians to identify areas of practice where their input could positively influence patients’ representations to aid with self-management and reduce psychological distress. The aim of the study was to explore the illness representations of patients with PsA who took part in focus groups to discuss important outcomes of treatment.

## Methods

A secondary analysis of eight focus groups held across five hospital sites (three in the Southwest of England, one in the West Midlands and one in West Yorkshire) and including 41 participants was undertaken. This study was approved by the National Research Ethics Service Committee North West-Haydock (reference 15/NW/0609) [10]. In the original study, data were collected between November 2015 and April 2016, and participants from a range of phenotypes and domains of disease activity were invited to participate: men = 20 and women = 21; mean (s.d.): age = 58(11.4) years; disease duration = 9 (8.3) years; and HAQ = 1 (0.7). Further details relating to the design, methods and analysis of the original study can be found in the paper by Dures *et al.* [10].

### Analysis

A deductive, theory-driven thematic analysis was conducted using the CSM as a framework [11, 12]. The aim of the analytical process was to support, extend or challenge the extent to which the CSM made sense of, and provided a comprehensive account of, participants’ PsA- and treatment-related representations. Data were read, re-read, coded for representations and then mapped onto the five illness representations of the CSM. The full data set was analysed by G.E., with a sub-set analysed by E.D. and S.H. (who collected and analysed the original focus group data).

## Results

The analysis provided evidence to support the existence of the five illness beliefs among patients with PsA, and evidence that these representations affected the way patients engaged with their condition. In particular, the evidence found that many participants experienced high levels of uncertainty in relationship to the identity, time line, cause, control and consequence of their PsA and its treatment. In addition to the five domains of the CSM, the role of external social and environmental factors also influenced participants’ illness representations and the way they made sense of their PsA. These illness representations appeared to be related to participants’ psychological state, including degree of distress. The data were mapped onto the five domains of CSM, using extracts to provide evidence for the presence of uncertainty and external factors and how these influenced psychological distress and the ways in which participants made sense of their PsA.

### Theme 1: identity

When discussing the identity of PsA, it became apparent that in addition to the physical symptoms there was an emphasis on the associated psychological distress. This distress was influenced by an uncertainty that participants perceived regarding the accuracy of their diagnosis, exacerbated by the diagnostic difficulties associated with PsA. The uncertainty surrounding the identity appeared to stem from a lack of communication between participants and clinicians, creating a barrier for participants to manage their PsA.

The data suggested that participants were significantly influenced by inter-individual, social factors, comparing themselves with others with PsA and members of the general population. When comparisons were made, they tended to be negative, with participants referring to themselves as inadequate or not normal, leading to low mood and depression. The invisible nature of the symptoms could also induce anxiety, with participants suggesting they felt like frauds. It was also highlighted that the general population’s understanding about the potentially disabling impact of PsA was often misinformed and inaccurate, possibly owing to its invisible nature. This caused some participants to experience problems with social participation and work, further contributing to the psychological burden of the condition.

**Table:** 

Theme	Sub-theme	Quote
Identity	Uncertainty	I’m still not convinced that that’s [PsA] what I've got … I’m not sure the label’s right. (Mic Pg10 FG5)
		I’m still not quite clear why it’s called psoriatic arthritis because I have very little psoriasis so … I’m not quite sure if the diagnosis is right. (Wil Pg5 FG1)
		And no one’s actually telling me what the problem is, and I can’t deal with it, because I don’t know what the problem is. (Ab Pg30 FG7)
	External	If anybody saw you they wouldn’t see that [diagnosis], so you feel sometimes like you are a fake. (So Pg9 FG2)
		People just don’t believe you. If somebody was to see us now they’d say what’s wrong with these guys? (Sid Pg22 Fg6)
		I get called selfish … so I have to constantly explain to my work, my wife, my children, my family, my friends why I’m not going out … the emotional side of that makes me quite insular. (Ma Pg 22 FG1)

Participant initial, page number and focus group number are provided in parenthesis for each quote.

### Theme 2: cause

Participants encountered difficulties and frustrations when it came to identifying the cause of the symptoms that they were experiencing. Participants were uncertain which symptoms to attribute to their PsA and which to their age, medications or other co-morbidities. Adding to the distress was the uncertainty that clinicians showed regarding the causality of symptoms. Participants also expressed uncertainty about the cause of their PsA because they had lived a healthy lifestyle, suggesting a lack of understanding about the condition.

The lack of understanding from the general population regarding the causes of PsA created another barrier for participants in social situations, including negative experiences from members of the public who thought that their PsA might be contagious. These encounters caused upset and distress.

**Table:** 

Theme	Sub-theme	Quote
Causal	Uncertainty	They don’t know what’s causing what… . (Nat Pg13 FG1)
		It strikes me that having the two problems [spinal/neurological and PsA/rheumatological] and not knowing what is what and how to deal with one and how to deal with the other. (Fl Pg31 FG7)
		But why should it happen to me when I’ve always looked after myself? (Am Pg18 FG8)
	External	I mean it’s people look at you and think, well, you know, is it catching? That’s the first thing people think … is, you know, can I get anything from that? (PL Pg38 FG4)
		I’ve had customers refusing to deal with me or shake my hand, or even use my pen … and it really, really upset me. (Ab Pg9 FG7)

Participant initial, page number and focus group number are provided in parenthesis for each quote.

### Theme 3: time line

In contrast to the other themes, participants showed certainty and awareness of the long-term and progressive nature of PsA, and this was the main source of fear and anxiety surrounding the management and prognosis. However, the unpredictable speed and intensity with which PsA might progress created uncertainty, although participants understood that clinicians were unable to offer predictions. The data also showed how participants considered themselves a burden and suggested they might have made different decisions if they had known the progression and outcome associated with PsA.

**Table:** 

Theme	Sub-theme	Quote
Time-line	Uncertainty	You worry what’s going to happen next, don’t you? How am I going to end up like? (Al Pg37 FG6)
		Obviously, nobody can tell us that we’re going to be here in 5 years. (Mar Pg32 FG7)
	External	You’ve got an added anxiety put on you by trying to treat your illness…. I genuinely wouldn’t have married him, if I’d have known it would get this bad, because it’s not fair. (St Pg12/14 FG3)

Participant initial, page number and focus group number are provided in parenthesis for each quote.

### Theme 4: consequence

The uncertainty surrounding the consequences of actions associated with PsA was demonstrated to be more changeable than other elements of the CSM. Participants explained that in the initial phases of diagnosis, a lack of knowledge and understanding about the consequences of PsA led to behavioural responses which, with hindsight, were not helpful. Examples include continuing in employment and leisure activities that exacerbated the condition. Uncertainty regarding the consequences and side effects of taking medications meant that some participants refused, or resented taking, pharmacological treatments. This could demonstrate a lack of understanding and misinformed illness representations, with potentially negative health outcomes for the patient.

The data also highlighted how negative illness representations could affect someone’s ability to take in and process information. This might be one explanation for the apparent lack of information provided by clinicians.

**Table:** 

Theme	Sub-theme	Quote
Identity	Uncertainty	I was diagnosed with psoriasis around 16, and being a typical bloke ignored it, didn’t take any notice. (Dar Pg1 FG6)
		My big bug bear is the damage caused … also … the lack of alteration in my behaviour…. I might have stopped mountaineering and marathon running if I had known I had arthritis. (Mil Pg4 FG8)
		I refused to take methotrexate … it gives you 99 bad things and one good thing. (Sid Pg23/24 FG6)
	External	Looking back now, I was just damn stubborn over it. I wasn’t listening to the professionals, and I wish I had done a lot sooner, because it [medication] did make a massive difference to me. (Ab Pg19 FG7)

Participant initial, page number and focus group number are provided in parenthesis for each quote.

### Theme 5: control

The management of PsA was a heavily discussed topic throughout all the focus groups, suggesting that symptom control and disease management were considered highly important among participants. However, the focus was on the medical and pharmaceutical management, and once again the findings showed significant uncertainty regarding the purpose and effectiveness of medications. This uncertainty, similar to theme 4 (consequences), was shown to have a negative impact on adherence. The lack of communication with clinicians or misunderstanding was once again highlighted as a major contributor to uncertainty and a potential barrier to disease management; for example, participants explained that they were unsure how to manage their PsA owing to insufficient information on what the medication was for and how it was working. Finally, the data suggested a gap in PsA management regarding the psychological support available. Several participants perceived that the clinician’s focus was only on their physical health, whereas their mental health was neglected and, as a consequence, they felt alone and at a loss.

**Table:** 

Theme	Sub-theme	Quote
Control	Uncertainty	I’ve got my doubts as to whether it’s [medication] working because I seem to be going backwards. (Ni Pg7 FG4)
		‘I don’t know if mine [medication] is working … they have given it to me for a reason, but I don’t know what it is supposed to be doing…. If I knew more about the medication then perhaps that would help me. I just don’t understand what the medication is actually doing’. (So Pg14 FG2)
	External	I felt very alone in the early days … there was never any information of what to look out for, or when you go to here. (Ad Pg15 FG3)
		You don’t really know what’s going on. So it’s just, take that, have that. (St Pg14 FG3)
		Everything is about your physical symptoms; there’s nothing about how to deal with the other side of things. [participant referred to impact on family life, relationships and emotional states] (Ma Pg24/25 FG1)
		I don’t think people recognize mentally what it does to you. (Mir Pg43 FG4)

Participant initial, page number and focus group number are provided in parenthesis for each quote.

## Discussion

The aim of this study was to explore the illness representations of patients with PsA. The findings of this study support the five domains of the CSM, with data from participants living with PsA fitting the framework. Two additional new findings to emerge in this current analysis are: (i) the high degree of uncertainty in relationship to participants’ representations; and (ii) the impact of external factors on participants’ illness representations. Furthermore, the data showed that the uncertainty and the external factors were contributing significantly to the psychological burden of PsA experienced by participants.

Although physical symptoms were a main feature of the focus group discussions, it was clear that the psychological impact of the condition was equally important. This supports previous studies that have demonstrated the negative impact of psychological distress on QoL, mental health and coping strategies in patients with PsA [2]. This includes research highlighting how some patients with PsA choose actively to avoid or hide from the burden of their condition, with suicide ideation being commonly expressed [3]. There is evidence to suggest that illness representations are an influential variable impacting on patients’ psychological distress [9]. By supporting patients to become more aware of their illness representations and the techniques they use to evaluate them, patients may be able to create more positive coping strategies [6]. Therefore, the finding in the present analysis that there is insufficient awareness of psychological impact and associated distress, with limited psychological support available, indicates the need for appropriate signposting or the development of services to support the emotional and social needs of people living with PsA.

One of the main findings, uncertainty, was present throughout the data and within each domain of the CSM, often demonstrated by the confusion, misunderstanding and vague responses expressed by participants. Uncertainty can be defined as the inability to determine the meaning of illness-related events and predict outcomes accurately, and it can take the form of ambiguity, complexity, lack of or inconsistent information, or unpredictability [13]. The uncertainty in illness theory proposes that uncertainty is influenced by the patient, the clinician and the social environment, and exists in situations of ambiguity [13]. Research has established that higher levels of uncertainty are related to poorer coping strategies and a greater sense of threat and distress [14]. This is supported in our present analysis, which found that the degree of uncertainty surrounding all elements of the condition created significant stress and anxiety among participants, impacting negatively on their psychological states, coping and physical health.

This uncertainty often appeared to stem from miscommunication and misunderstanding between clinicians and patients, which may have contributed to the development of negative illness representations and, subsequently, to poor coping strategies and self-management. The findings show that where participants were uncertain regarding the diagnosis, outcome of treatment and controllability of symptoms, their adherence to treatment regimens was hindered. As suggested by Hagger and Orbell [7], where illness is perceived as highly symptomatic then chronic avoidance and emotion-based coping strategies are more likely to be adopted. In contrast, an improved understanding of disease management and symptom control can positively impact on psychological states and social interaction [7].

Illness representations and uncertainty are both shaped by an individual’s external social environment [7, 13]. The findings of the present study highlight how participants were highly influenced by the opinions of, and interactions with, friends, family and clinicians, in addition to the general population. The data showed how several participants felt like frauds and burdens on friends and family, demonstrating the impact of their perception of other people’s thoughts and feelings about them, in addition to their own thoughts about their PsA and how the associated restrictions affected others. Research in RA has shown that social context has a significant impact on how individuals deal with stress and anxiety [15]. Similar to the findings of the present study, Bediako and Friend [15] found that the expectations of misinformed family and friends in relationship to the patient’s ability to function and manage their condition were frequently misaligned with the patient’s own perceived capabilities, causing stress and anxiety.

Finally, although participants’ illness representations can be mapped within the CSM, it could be suggested that these specific beliefs are flexible and exist only at the point of assessment, because illness representations have been shown to be influenced by multiple factors. Few research studies have examined illness representations using longitudinal designs, and in the present study data are cross-sectional. However, participants’ accounts of how their beliefs changed over time suggest the potential for illness representations to be flexible rather than stable. There is some evidence from other long-term conditions to support this. These changes can start in response to diagnostic results and have been associated with emotional distress, recovery and disability, and with treatment-related behaviour such as adherence [16]. Thereafter, the changes can continue. For example, research in maintenance haemodialysis found that over a 6-year period, patients had fewer negative emotional reactions, understood their disease better, considered dialysis more efficient in controlling their end-stage renal disease and perceived their illness as having a long-term course [17]. Likewise, in diabetes, researchers found that patients’ emotional representations decreased within 2 years of diagnosis, whereas illness coherence increased [18]. A 6-year study in OA also found that illness representations changed over time, and these changes were related to outcome, in particular the progression of disability. Moreover, illness perceptions were predictive of disability. This might imply that interventions aimed at changing illness representations can contribute to better functional outcome [19]. This has important clinical implications and suggests that future research should focus on which illness representations are amenable to change in patients with PsA, the timeliness of interventions and the processes by which helpful changes might be facilitated. Finally, research has also demonstrated in patients with coronary heart disease how over the course of 1 year the QoL and global health status declined, with the disease severity and history accounting for only half of the change and illness representations accounting for the other half [20]. This highlights how an alterable aspect of disease progression, illness representations, could be targeted during treatment to reduce the decline in QoL and health status.

### Limitations

This was a secondary analysis of focus group data, which had been collected to explore the issue of patient priorities for treatment. Consequently, participants’ illness representations were not specifically or explicitly investigated. If insights into the illness representations of patients with PsA had been the aim of the focus groups, researchers would have had the opportunity to elaborate or unpick points of interest relating to the CSM. However, it is of interest that the nature and influence of illness representations were manifest without being prompted, emphasizing their importance in how participants were responding to their PsA and its treatment. A further limitation is that focus group participants were recruited from the UK only. As participants’ uncertainty appeared, at least in part, to be related to information and communication with clinicians, and external factors were also an important feature, these findings might have limited transferability to different health-care settings and social and cultural contexts.

### Implications

A greater understanding of the high levels of uncertainty that patients might have about their condition and its treatment could provide clinicians with an opportunity to address misinformed or erroneous illness representations and ambivalence about treatment. Supporting patients to acquire reliable information and express their concerns has the potential to improve QoL and facilitate greater self-management of their PsA. Furthermore, by actively engaging patients in discussions regarding their psychological health, including the impact of external factors on their well-being, clinicians would be able to identify appropriate support pathways and ensure that any further referrals that might be needed are made.

The Programme grants for applied research, early detection to improve outcome in patients with undiagnosed psoriatic arthritis (PROMPT) Study Group comprises the following non-contributing authors: William Tillett (Royal National Hospital for Rheumatic Diseases, Bath and the University of Bath), Gavin Shaddick (University of Exeter), Alison Nightingale (University of Bath), Helen Harris (Number 18 Surgery, Bath), Philip Helliwell (University of Leeds), Laura Coates (University of Oxford), Catherine Fernandez (University of Leeds), Claire Davies (University of Leeds), Sarah Brown (University of Leeds), Jon Packham (University of Keele), Laura Bojke (University of York), Eldon Spackman (University of Calgary), Catherine Smith (Guy’s and St Thomas’s NHS Trust and Kings College London), Anne Barton (University of Manchester), Oliver Fitzgerald (University of Dublin), Vishnu Madhok (Park House Surgery), Melanie Brooke (Patient Research Partner), Jana James (Patient Research Partner), Andrew Parkinson (Patient Research Partner).
